# Physical Limitations of Phosphor layer thickness and concentration for White LEDs

**DOI:** 10.1038/s41598-018-20883-3

**Published:** 2018-02-05

**Authors:** Cher Ming Tan, Preetpal Singh, Wenyu Zhao, Hao-Chung Kuo

**Affiliations:** 1 Centre for Reliability Science and Technology, Chang Gung University, Wenhua 1st Road, Guishan Dist., Taoyuan City, 33302 Taiwan; 2 Department of Electronics Engineering, Chang Gung University, Wenhua 1st Rd., Guishan Dist., Taoyuan City, 33302 Taiwan; 3 Department of Urology, Chang Gung Memorial Hospital, Guishan, Taoyuan 333 Taiwan; 4 Department of Mechanical Engineering, Ming Chi University of Technology, 84 Gungjuan Rd., Taishan Dist., New Taipei City, 24301 Taiwan; 5 Inner Mongolia University of Science and Technology, School of Chemistry and Chemical Engineering, Baotou, 014010 China; 6 Department of Photonic and Institute of Electro-Optical Engineering, National Chiao Tung University, Hsinchu, 300 Taiwan R.O.C

## Abstract

Increasing phosphor layer thickness and concentration can enhance the lumen flux of white LED (W-LED). In this work, we found that increasing the phosphor layer thickness and concentration can increase its temperature, and there is also a maximum thickness and concentration beyond which their increase will not lead to lumen increase, but only temperature increase. Higher thickness and higher concentration also results in warm light instead of White light. The maximum thickness and concentration are found to be limited by the scattering of light rays with higher % decrease of blue light rays than the yellow light rays. The results obtained in this work can also be used to compute the temperature and thermo-mechanical stress distribution of an encapsulated LED, demonstrating its usefulness to the design of encapsulated LED packages. Simulation software like ANSYS and TracePro are used extensively to verify the root cause mechanisms.

## Introduction

Increased lifetime and efficiency led to rapid expansion of LED’s application areas^1–5^, but the cost of high power white LED (W-LED) is still on the high side. Hence works are done to increase the lumen flux of W-LEDs so as to reduce the number of LEDs required for a given illumination level, and helps in cost saving. Increase efficacy of W-LEDs can also reduce the power consumption for a given illumination, and results in further cost saving^6,7^.

Common cost effective ways to achieve the increased lumen flux and efficacy are to use thicker phosphor layer and/or higher phosphor concentration to increase the efficacy^8–11^, and higher current to drive the LEDs to increase the lumen flux^12,13^. Use of remote phosphor technology^14–19^ and placement of phosphor in different shapes to increase the light output and reduce the effect of heat generated by phosphor on the blue LED dice during light conversion process^20–24^ are other ways.

While these methods can indeed achieve the intended goals, the physical limitations of these methods have not been investigated. Also, the reliability consequences of the abovementioned two methods are not addressed due to several reasons as discussed in literatures^1,5,25,26^. For the case of remote phosphor technology, it has been shown that this method can cause an increase in the phosphor temperature upto 40 °C or in some cases 60 °C due to the lack of effective heat dissipation path, and this leads to rapid discoloration and overall light output degradation^27,28^.

It is the purpose of this work to examine the effects of increasing phosphor layer thickness and concentration on the luminous flux, CIE color coordinates and their reliability. The effect of increasing the drive current will be published elsewhere. An application of our method to the design of encapsulated LED is also demonstrated.

## Experimentation

**Figure 1:**
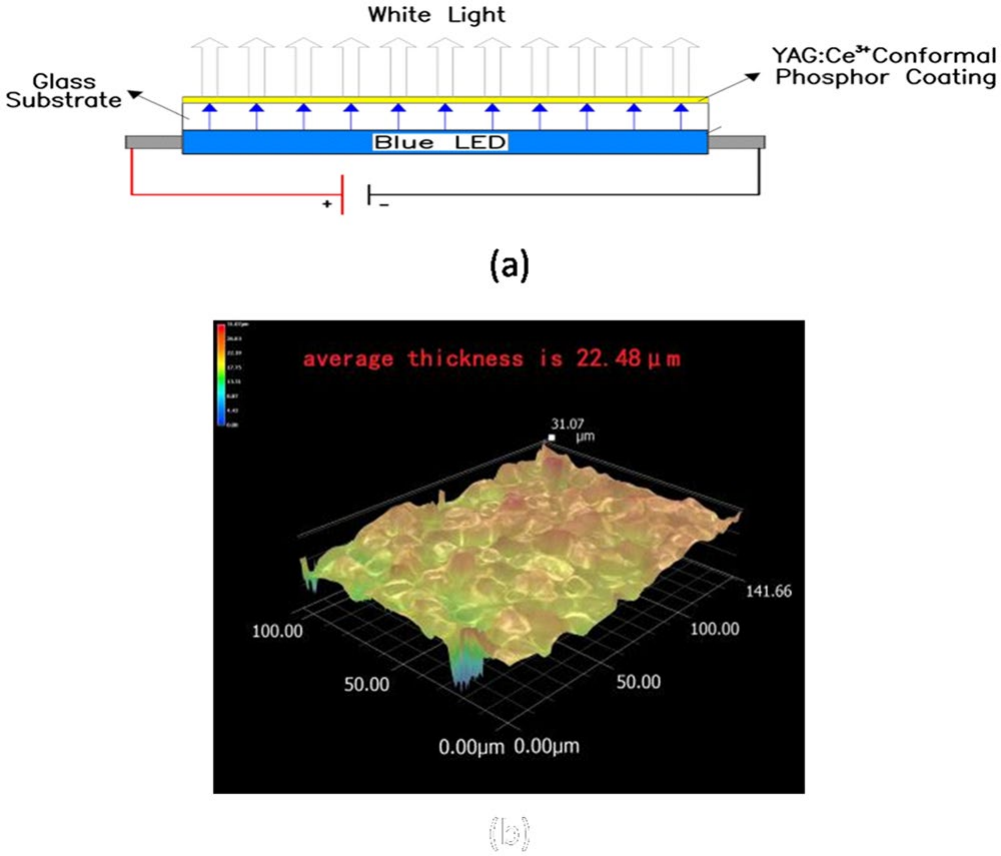
(**a**) Experimental Set –up for this work. (**b**) Optical Micrograph of a phosphor coating on glass slide.

OSRAM high power blue LED and INTEMATIX phosphor coating layer on a glass substrate are used in this work. Ce is doped into our phosphor via sol- gel combustion synthesis method which is a mature technology that provides a uniform doping of Ce into phosphor throughout the samples, and it is commonly used in industry^29–31^. Borosilicate glass slide is placed on top of the blue LED to obtain white light from the blue LED as shown in Fig. 1(a). Pulsed spray process is used for the conformal phosphor coating YAG: Ce^3+^ on the glass slide as substrate with four different thicknesses of coating, keeping the concentration of the phosphor particles constant. The average coating thicknesses are 14.12, 22.48, 30.53 and 36.52 μm respectively. Figure 1(b) shows an optical micrograph of the phosphor coating of 22.48 μm at a magnification of 2500× using Keyence microscope VHX5000. Optical properties and temperature of the phosphor layer are measured using integrating sphere Ocean Optics QE65000 and infrared thermal imaging system P384A3-20 respectively.

Samples of different phosphor thicknesses with the set-up shown in Fig. 1(a) are placed in the integrated sphere, and different constant currents are supplied to the blue LED using Agilent power supply model E3630A that is connected to the sphere, for optical properties measurement. For the phosphor temperature measurement, these samples are placed outside the sphere and the blue LED is driven by constant currents from Keithley power supply model 2651 A. Their temperatures are measured using infrared thermal imaging system P384A3-20.Keyence VHX5000 high magnification 3D confocal microscope is used to observe the surface morphology and measure the thickness of the phosphor layer coated on the glass surfaces. SEM model S-2600H along with EDS model RONTEE is used to identify and observe the presence of phosphor layer on the glass surfaces.

## Results and Discussion

**Figure 2:**
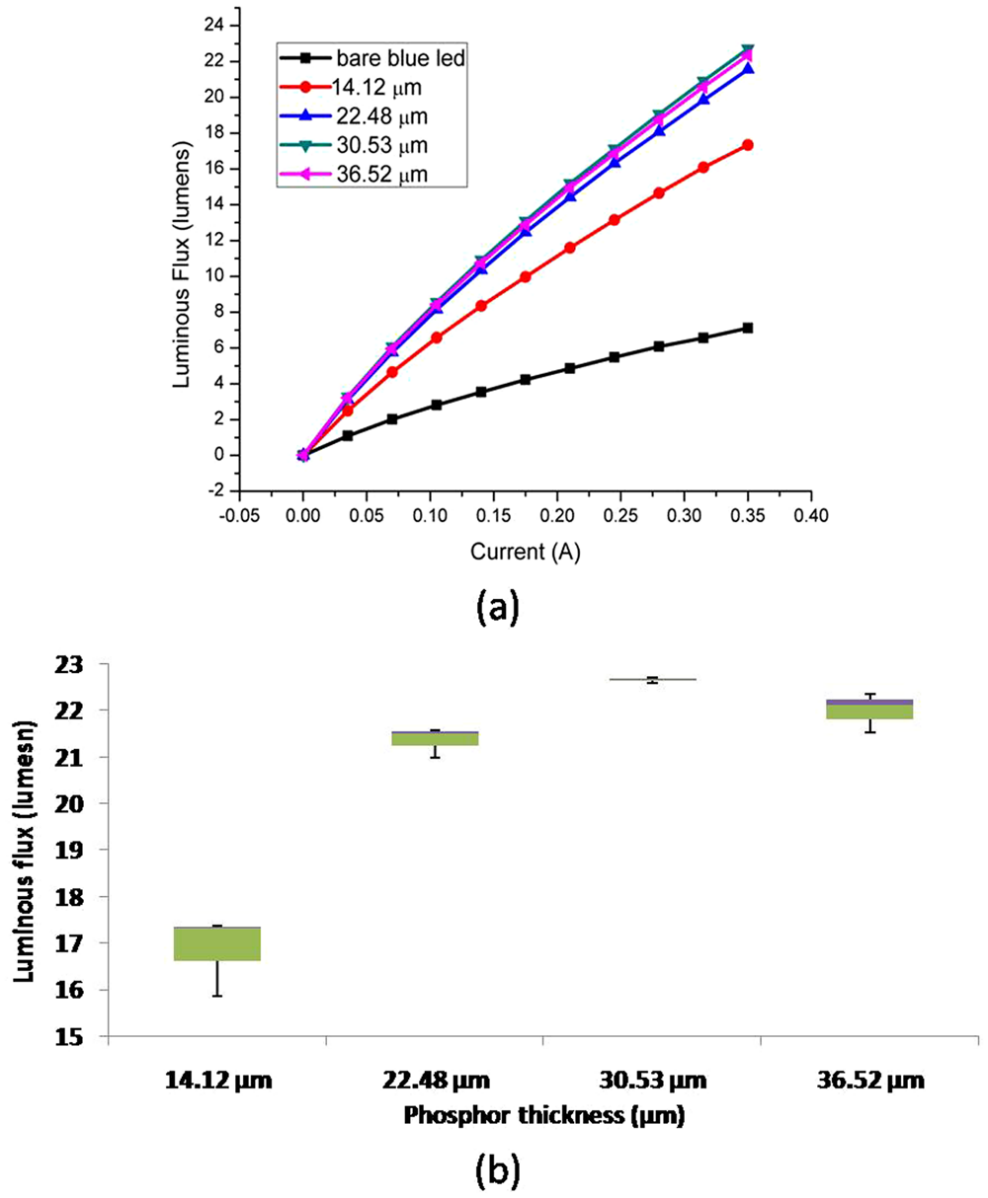
(**a**) Total Lumen flux of the set-up for different phosphor layer thickness at various amount of drive current. (**b**)Box plot demonstrating the luminous flux for 3 samples used at each phosphor layer thickness at 350 mA to verify the luminous flux variation at different phosphor thickness.

YAG:Ce^3+^ phosphor converts part of the blue light pumped by the blue LED into yellow light, and the combination of the remaining blue and yellow light produces white light, resulting in W-LED^32,33^. Hence, luminous flux will be higher for LEDs with more light-converting phosphor particles, resulting in higher luminous efficacy as shown in Fig. 2.

**Figure 3:**
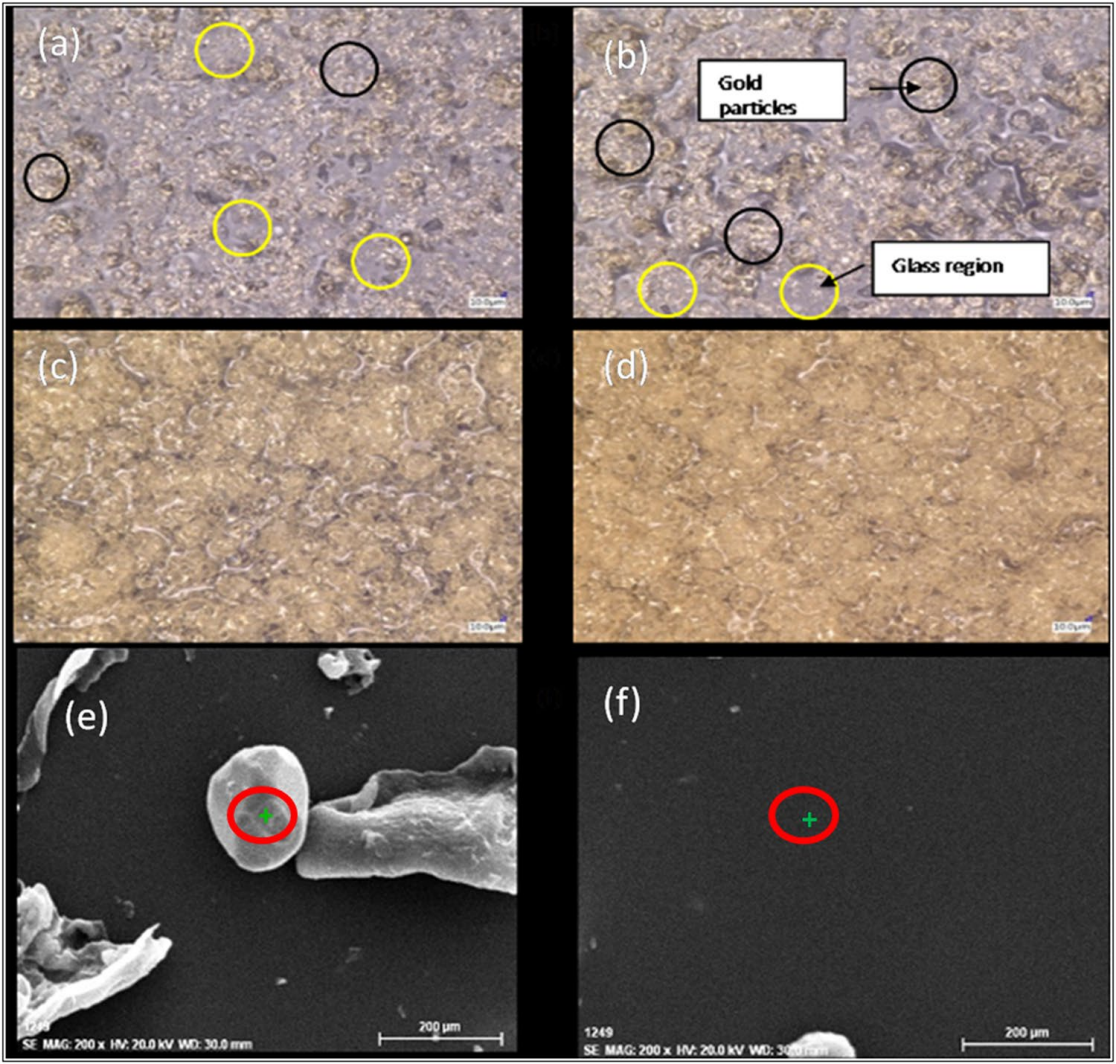
Optical micrograph of the phosphor layers on glass slides. (**a**–**d**) Are for the phosphor layer thickness corresponding to 14.12, 22.48, 30.53 and 36.52 μm respectively. In (**a**) and (**b**) black outlined boxes show the gold color phosphor particles and the yellow outlined boxes show the grayish glass surface. (**e**) Is the SEM micrograph of a black outlined box for the confirmation of the phosphor particle and (**f**) is the SEM micrograph of a yellow outlined box for the confirmation of the glass area. The “+” symbols in (**e**) and (**f**) are the locations of EDS analysis where the results are shown in Table 1.

Figure 2(a) shows that the lumen flux due to the increase in the phosphor layer thickness at constant phosphor particles concentration is larger with higher current, but the amount of increase in the lumen flux with the phosphor layer thickness decreases at higher thickness despite an increase in the total amount of phosphor particles. In fact, the lumen flux has a slight decrease when the thickness increases from 30.53 to 36.52 μm as shown clearly in Fig. 2(b) which is a Box plot of our results where 3 samples are used at each phosphor thickness. The slight decrease of the light intensity for thickness beyond 30 μm is unlikely due to the droop effect of the blue LED itself as this can be evident from Fig. 2(a). Bare blue LED luminous flux vs current data in Fig. 2(a) verifies that there is no droop effect of the blue LED itself in the data as there is no luminous flux decrease observed at higher currents. <how the optical micrographs of the samples, and the gold color structures in the Figure are the phosphor particles in spherical form as confirmed using SEM and EDS as shown in Fig. 3(e–f) and Table 1. EDS results show that the particles on the glass consists of Yttrium (Y), Aluminum (Al), Cerium (Ce) and Oxygen (O), whereas the rest of the surface consists only Silicon (Si), Oxygen (O) and Carbon (C) as shown in Table 1. Gold is sputtered on the surface of the samples to inhibit charging, reduce thermal damage and improve the secondary electron signal required for topographic examination in the SEM.

**Table 1:** EDS results of the phosphor particle and the glass area. EDS results are obtained using EDS model RONTEE at an accelerating voltage of 20 keV.

Phosphor particle(in Fig. 3(a))		Glass area (in Fig. 3(b))	
Element	Atomic Concentration [wt. %]	Element	Atomic Concentration [wt. %]
Oxygen	11.21	Carbon	22.66
Aluminum	11.54	Oxygen	25.83
Yttrium	51.85	Silicon	40.82
Gold	13.15	Gold	10.68
Cerium	12.21		

It is clearly seen that when the phosphor layer thickness is small, the coverage of the phosphor layer over the glass is much less than 100%, and hence the yellow light intensity is small as shown in Fig. 3(a–d). As the phosphor layer thickness increases, the coverage also increases, and correspondingly the yellow light intensity.

**Figure 4:**
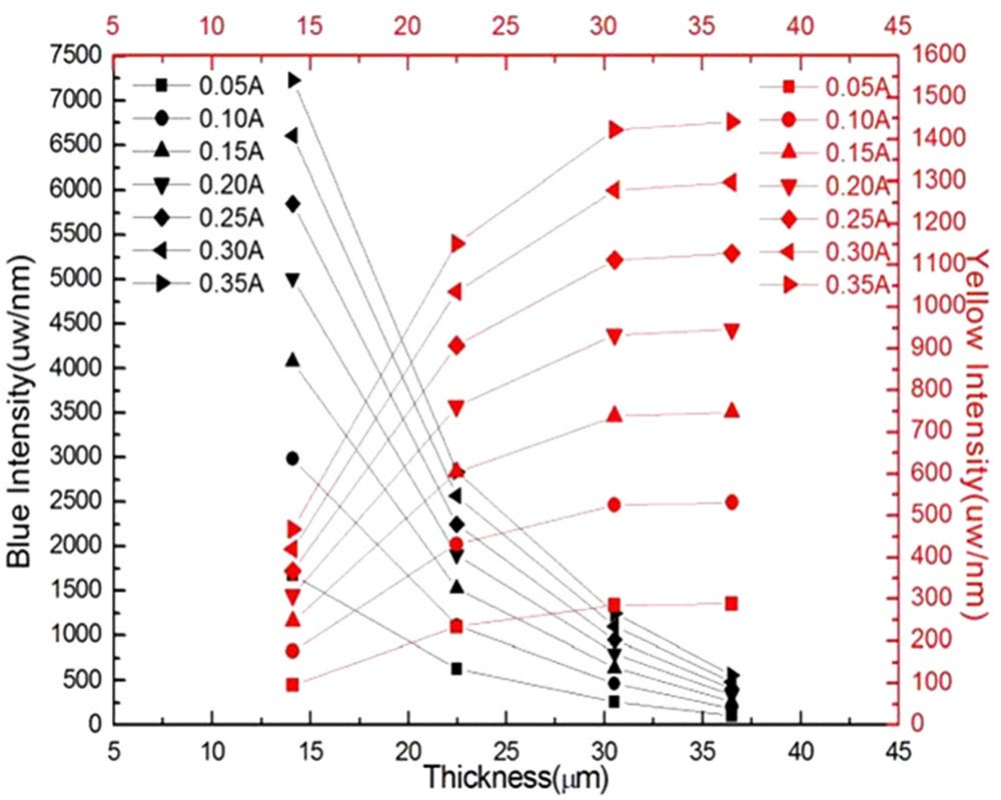
Radiant flux of phosphor coating layers with different thickness at a range of 350 nm–850 nm. The blue intensity is obtained at 450 nm, and the yellow intensity at 590 nm.

Figure 4 shows the radiant flux of phosphor coating layers with different thickness at a range of 350 nm–850 nm. One can see in Fig. 4 that the blue light intensity decreases as more blue light is converted to yellow light as expected, but when the phosphor layer thickness goes above 23 μm, the rate of increase in the yellow light intensity with respect to the increase in the phosphor layer thickness is decreasing, and likewise the rate of decrease in the blue light intensity with respect to the increase in the phosphor layer thickness, even though the total amount of phosphor particles is increasing. This trend is regardless of the drive current. These observations could be due to surface roughness reflection, self-absorption^34^, phosphor sedimentation^35^, scattering of light or back reflection by the densely distributed phosphor particles. Let us investigate each of these possible causes in detail.

**Figure 5:**
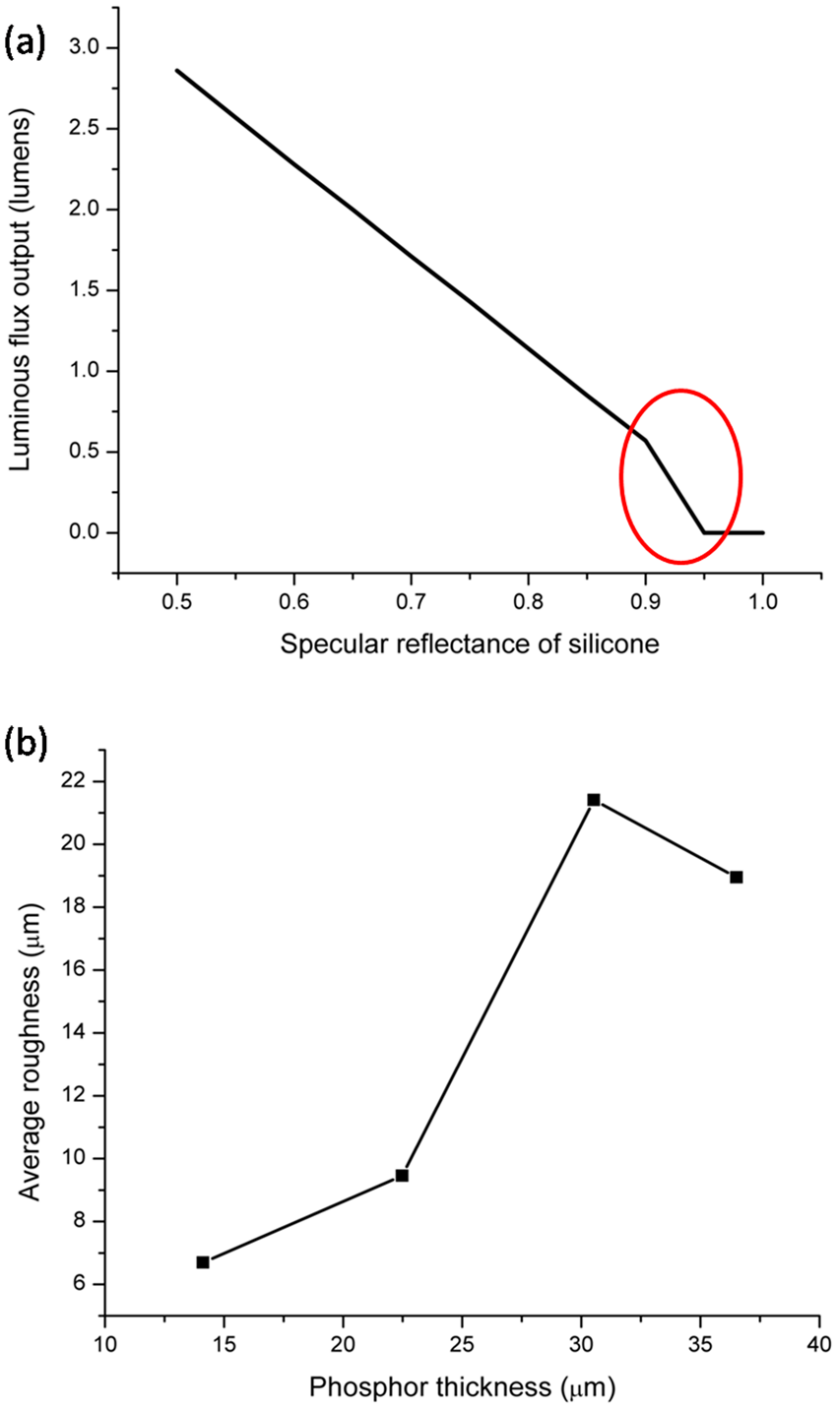
(**a**) Effect of specular reflectance at silicone surface on the luminous flux output in LEDs as computed using TracePro software. When the specular reflectance is 1, it represents a perfect smooth surface. Smaller value of specular reflectance corresponds to rougher surface. (**b**) Average roughness of the phosphor layer for samples with different phosphor thicknesses. The measurements are done with 3D confocal optical microscope Keyence VHX-5000.

TracePro, an optical rays simulation software, is employed to observe the effect of surface roughness at the phosphor-air interface as shown in Fig. 5(a). If the surface of a phosphor layer is smooth, more light will be reflected back from the surface to the inner part of the phosphor layer due to the difference in the refractive indexes of phosphor and air, render lesser amount of light going out of the phosphor layer as shown in Fig. 5(a). The luminous flux output from LEDs is almost zero for specular reflectance from 1 to 0.95 as most of the light is reflected back, and for specular reflectance below 0.95, we can see a rise in luminous flux. This can be seen as a sharp change in Fig. 5(a) for specular reflectance in the range of 0.9 to 1. In our samples, surface roughness of phosphor layer actually increases with its thickness as shown in Fig. 5(b). The increase in roughness is larger for thickness beyond 23 μm, and thus the rate of increase in the luminous flux output should be larger, according to the roughness effect expected from the TracePro simulation, but this is against our experimental observations. On the other hand, for thickness beyond 30 μm, the roughness starts decreasing and the observed luminous flux also starts decreasing as observed. In other words, surface roughness change cannot be used to explain our experimental observations. However, surface roughness might be a possible reason for the lumen reduction for the part where the phosphor layer thickness is beyond 30 μm.

Most phosphor used in LED are broadband and based on either Ce^3+^or Eu^2+^. These broadbands lead to overlap between emission and absorption profiles in these phosphors, and often leads to the possibility of self absorption^34^. The self absorption process take place by non – radiative transfer of energy or by actual emission and re-absorption of photons. The self absorption process could lead to many interseting effects such as peak shape changes and/or red shifts^34^. In our work, Ce3+ is used for phosphor, and hence there is a possibility of self absorption or re-absorption which can lead to spectral changes as mentioned above and temperature rise, and also decrease in the lumen flux output.

**Figure 6:**
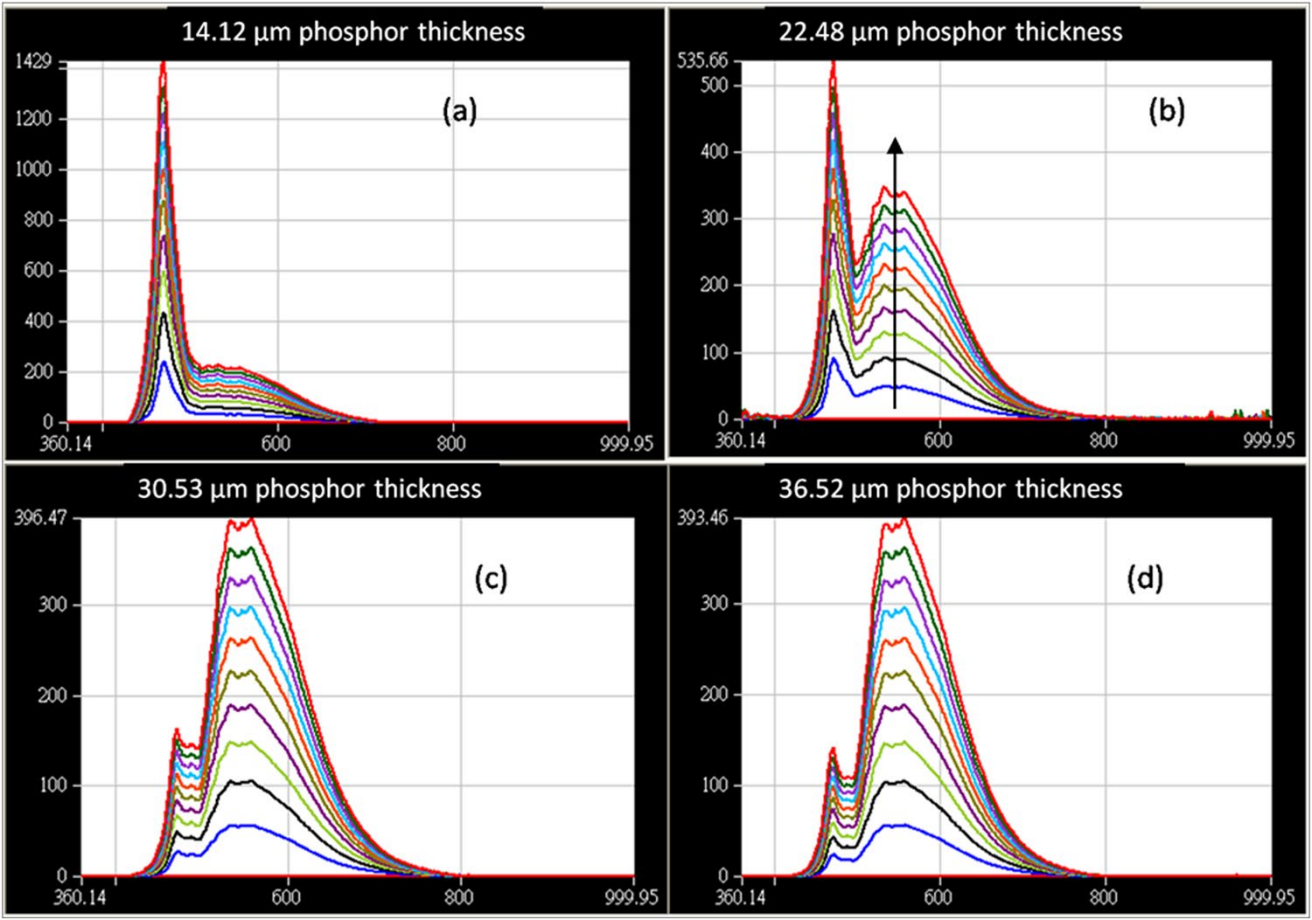
Emission spectra for the light output at different phosphor thickness (**a**) 14.12 μm, (**b**) 22.48 μm, (**c**) 30.53 μm and (**d**) 36.52 μm. The arrow in the figure indicates increasing drive current of the blue LED.

Figure 6 shows the emission spectra of our samples at various amount of drive current up to 350 mA, and one can see that the shape of the spectra and the peak wavelength do not show any significant red shift for the yellow peak. All are situated at 557.15 nm. This suggests that there is negligible self-absorption phenomenon occur at the phosphor sites in our samples, and self- absorption is not the reason for the light loss or temperature rise in this work.

The concept of phosphor sedimentation is introduced by Hu *et al*.^35^. As phosphor particles are dispersed in silicone medium, and due to the weight of the particles, they will settle to the bottom, resulting in changing concentration of the phosphor particles, being the highest at the bottom and gradually decreases toward the top. Consequently, the refractive index within the phosphor layer also changes gradually, and internal reflection within the phosphor layer can occur.

**Figure 7:**
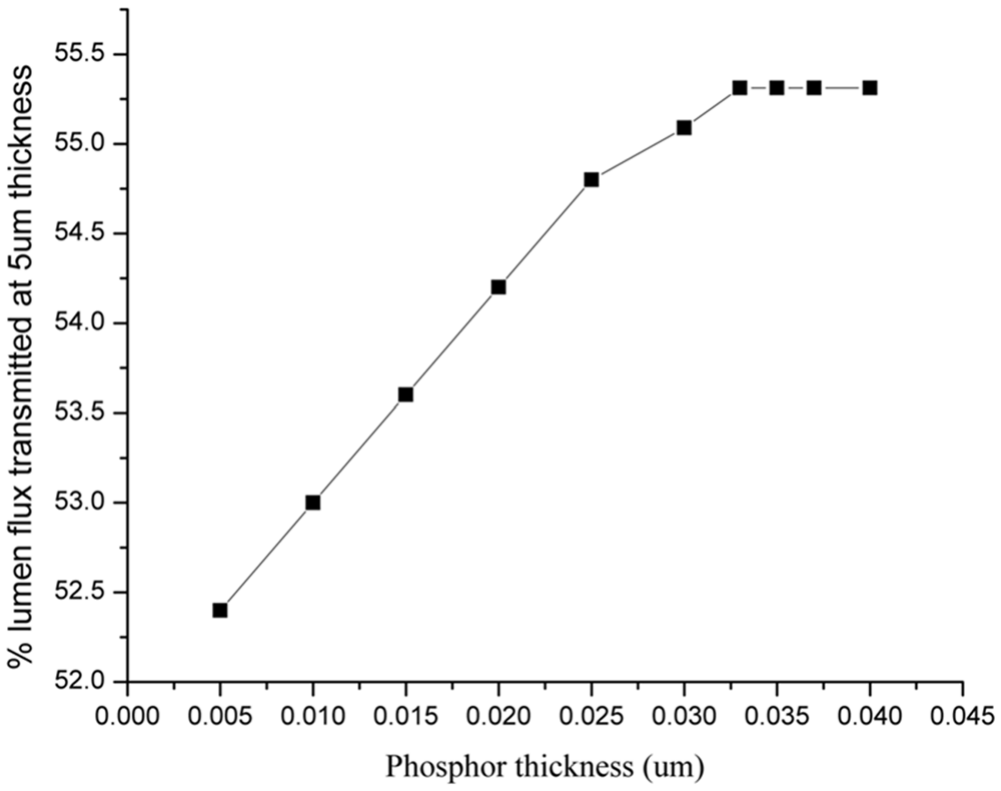
TracePro simulation of the % of blue light transmitted out of the phosphor layer with the phosphor thickness due to phosphor sedimentation.

To examine if the phosphor sedimentation could be the mechanism of the above-mentioned observations, TracePro is employed again. A simple structure is built where the % of phosphor particles is changing stepwise from 100% at the bottom 5 μm with a step of 20% reduction across 5 μm thick of phosphor layer, and the profile of phosphor content % is shown in the insert of Fig. 7. This profile is set arbitrary for the purpose of examining the effect of the sedimentation.

With this profile, the refractive index also changes, and the refractive index of a phosphor segment is a weighted average of the refractive index of phosphor particles and the silicone medium (the refractive index of silicone is 1.4188, and that for phosphor is 1.81^36,37^). For example, for the layer with 80% phosphor particles, the composite refractive index is equal to 0.8 $\ast$ 1.81 + 0.2 $\ast$ 1.4188 = 1.72.

For simplicity, only blue light is considered. Also, conversion of blue light into yellow light through phosphor particle and surface roughness at the interface of phosphor layer and air are not considered in the simulation. The change of refractive indexes with temperature is not considered as well, even though the temperature of the phosphor layer is increasing with the thickness of the phosphor layer as will be shown later in this work.

Figure 7 shows the % of lumen flux transmitted out of the phosphor layer vs the phosphor thickness with our simple model. We can indeed see the decreasing rate of the light output intensity with the phosphor layer thickness and a plateau is observed beyond 30 μm of phosphor layer as very few phosphor particles reside and it leaves mainly silicone at the top segment.

This sedimentation seem to explain the surface roughness profile shown in Fig. 5(b). The drop in the roughness after 30 μm of phosphor could be expected with the phosphor sedimentation phenomena. If most of the phosphor particles are sediment up to the thickness of 30 μm, then the region above 30 μm will mostly be the silicone medium, and it will be smoother as very few phosphor particles are on the surface of the interface between the silicone and the air, in analogy to the sedimentation of sandy water.

As smooth surface tends to reflect more light back to the phosphor as seen earlier in Fig. 7, the drop in the lumen light output could be due to the surface reflection as phosphor sedimentation could not explain the plateau of the light reflection after 30 μm. This seems to be consistent with Fig. 4 where the blue light output decreases without an increase in the yellow light when the phosphor thickness is above 30 μm, indicating that the blue light is reflected back into the phosphor layer.

**Figure 8:**
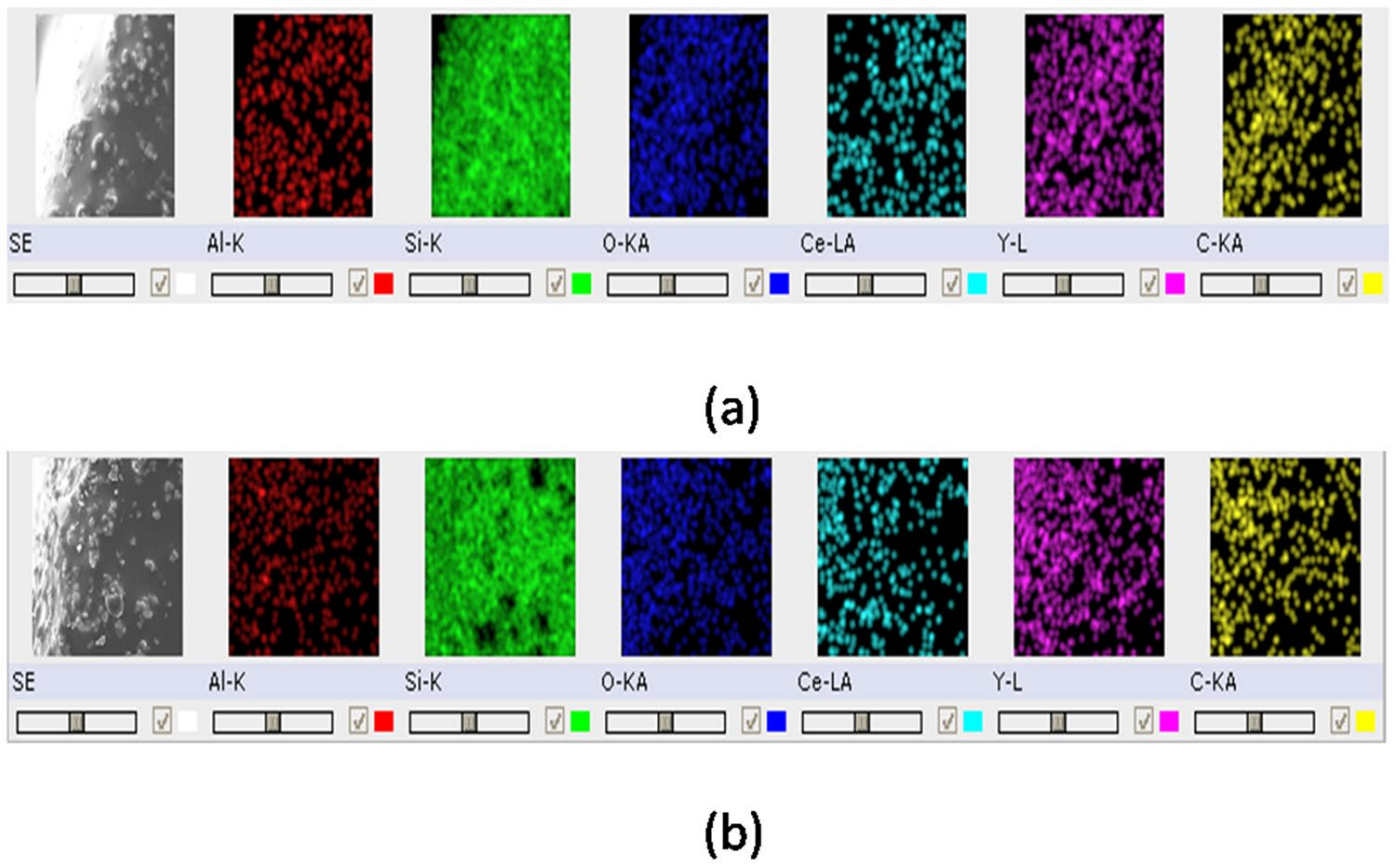
Observation of homogenous distribution of phosphor particles in silicone using EDS mapping for samples with phosphor thickness of (**a**) 30.53 um and (**b**) 36.52 um respectively.

To verify the phosphor sedimentation phenomenon, cross-section of the phosphor layers is made, and EDS is employed to obtain the elemental mapping of samples with varying thicknesses. EDS is done at an accelerating voltage of 20 kV and the SEM is set to 500× magnification. EDS mapping for samples with phosphor thickness of 30.53 um and 36.52 um are shown in Fig. 8 as the sedimentation effect should be higher in these samples due to higher amount of phosphor particles, and that the much lower amount of particles near the surface due to sedimentation should be observed.

However, from the EDS mapping, we see that Ce, Al and Y which constitutes the YAG phosphor are randomly or homogenously distributed throughout the thickness. This violates the proposal of phosphor sedimentation. In fact, various techniques are employed today to avoid sedimentation and agglomeration of phosphor particles, in order to ensure homogenous distribution of phosphor particles in the composite^38,39^ and our phosphor layer is indeed using one of these techniques.

**Figure 9:**
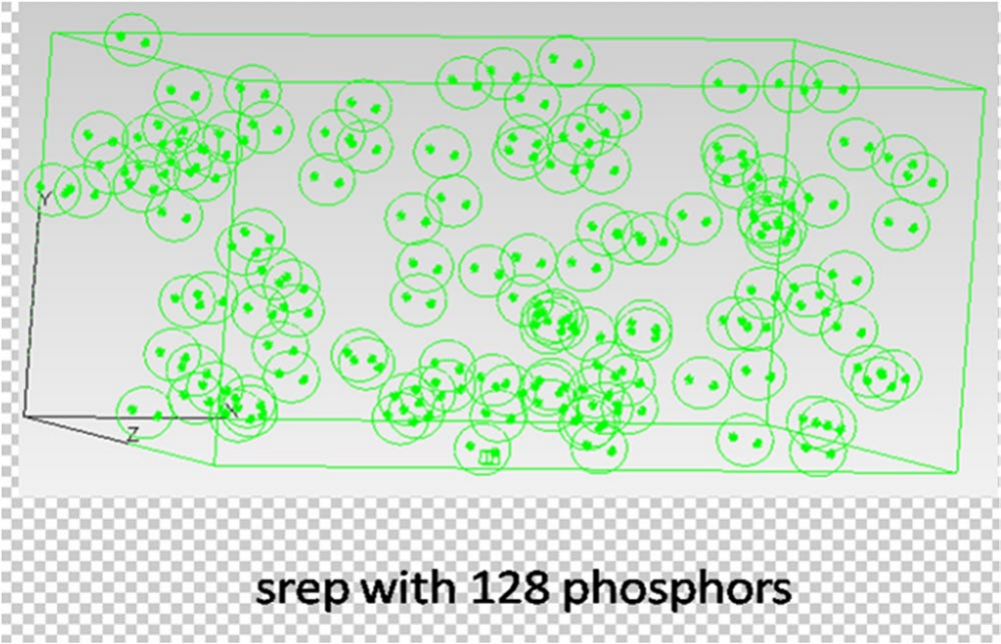
TracePro structures with 128 phosphor particles.

Light scattering and reflection from densely distributed phosphor particles is the last remaining possible mechanism. We employed TracePro and ANSYS for the investigation. In order to examine the separate effect of phosphor layer thickness and amount of phosphor particles, we first examine the effect of the amount of particles while keeping the phosphor layer thickness constant. Five different structures with varying number of phosphor particles are used and the structure is shown in Fig. 9 for 128 phosphor particles. Phosphor particles position is randomly generated to ensure homogeneity. Since we are not able to obtain the actual number of phosphor particles inside the silicone-phosphor composite, the sets of 32, 64, 96, 128 and 160 phosphor particles are generated in the silicone matrix for qualitative investigation.

The chromaticity coordinates of the phosphor material’s emission, absorption, and excitation energy are determined by its relative emission, absorption and excitation spectra as done previously by Chao-his Tsao *et al*. in their work^40^. The parameters used for phosphor in the TracePro simulation are shown in Table 2.

**Table 2:** Simulation parameters used in TracePro for silicone and phosphor.

	Phosphor	Silicone
Quantum efficiency	0.92	
Peal Molar Extinction [liter/(mole*cm)]	1000	
Temperature (K)	300	300
Thickness [um]		30
Refractive index	1.82	1.4
Radius (um)	2	

**Figure 10:**
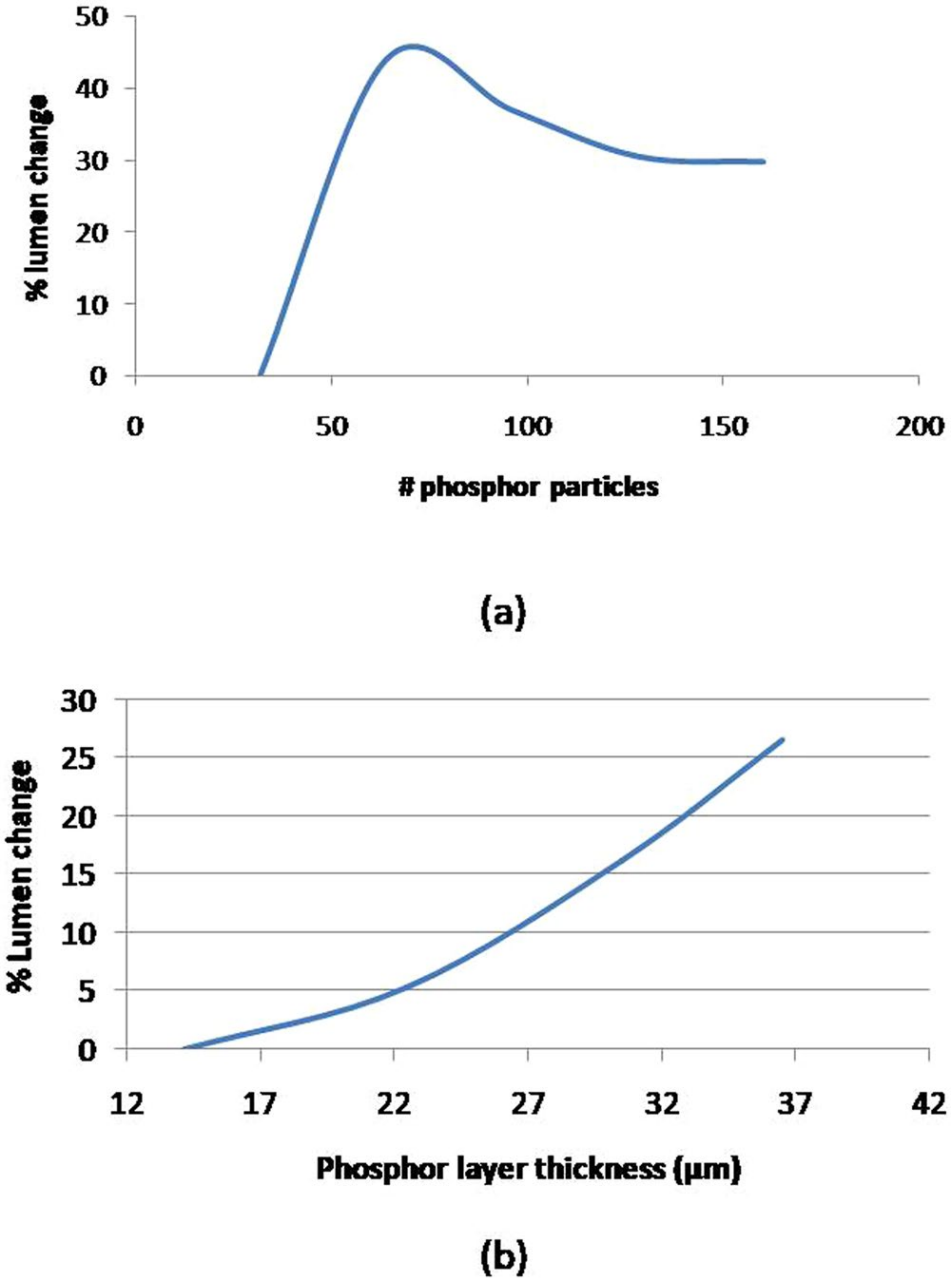
TracePro simulation results. (**a**) % Lumens change due to different amount of phosphor particles with 32 phosphors particles as reference. (**b**) % Lumen change due to increasing phosphor layer thickness while the number of phosphor particles is kept at 64.

TracePro results for different samples are presented in Fig. 10(a) where the % change in luminous flux is presented. From Fig. 10, it is observed that there is a rise in lumens as the number of phosphor particles increases from 32 to 64, and a fall in lumen value is observed after 64 phosphor set. This result seems to be in accordance with our experimentation results qualitatively, indicating that the amount of phosphor particles will indeed cause an increase and followed by a decrease in the output lumen. The physical explanation will be given later.

On the effect of thickness for a given number of particles, we perform similar simulation of varying phosphor thickness with 64 phosphor particles as the lumen value is the highest for 64 phosphor particles during our initial simulation, and the result is as shown in Fig. 10(b).

**Figure 11:**
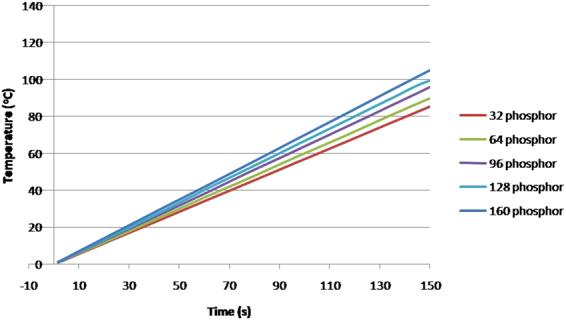
The temperature rise comparison between different samples with varying phosphor particle numbers.

The results shown in Fig. 10 can be explained from the light scattering and back- reflection principle. In Fig. 10(a), the transmitted light increase with increasing phosphor concentration is expected as higher amount of blue to yellow light conversion process is taking placing. This is consistent with the observation shown in Fig. 6 where the blue light intensity is decreasing and yellow light intensity keep on increasing till 30 µm. As the efficiency of the conversion process is not 100%, the heat generated during the conversion also increases with more phosphor particles, and this is revealed as a continuous increase in the temperature of the phosphor layer as shown in Fig. 11.

**Figure 12:**
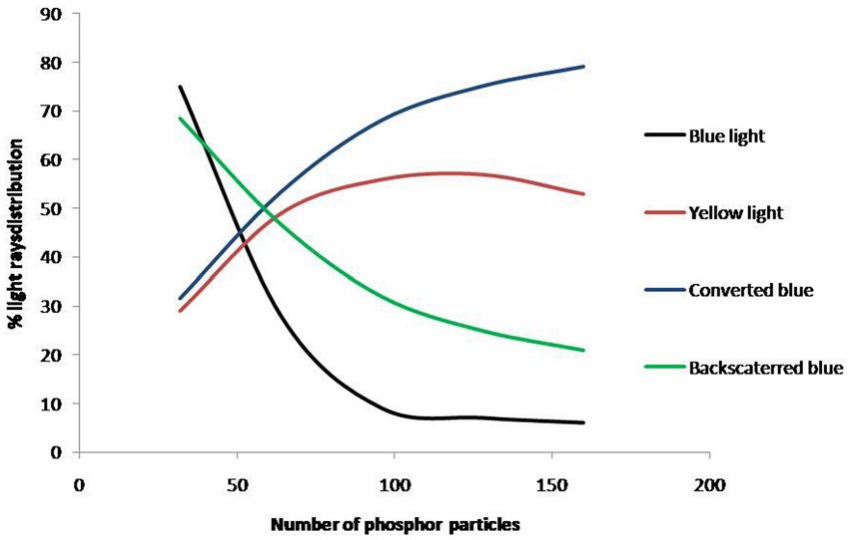
Blue and Yellow light rays distribution with varying number of phosphor particles. The black line represents the % of blue light rays out of the silicone. The blue line represented the % of blue light rays participated in the conversion to yellow light, which form part of the total decrease in the blue light rays. The green line represents the % of blue light rays scattered backward due to phosphor particles. The red line represents the ratio of the total yellow light rays emitted out of silicone expressed in %.

**Figure 13:**
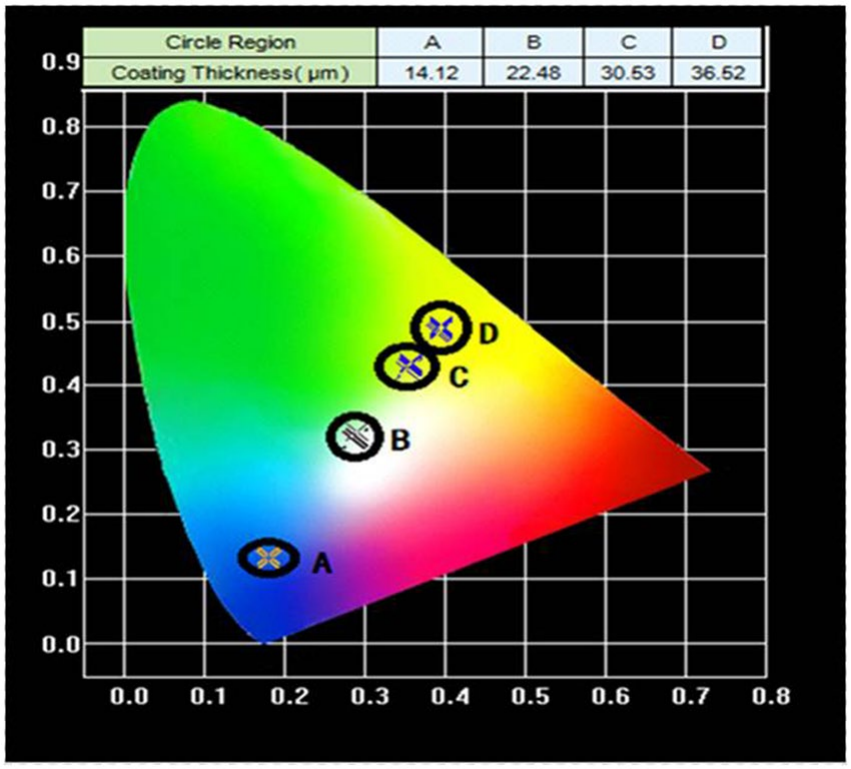
(**a**) CIE diagram of phosphor coating with different thicknesses at drive current of 350 mA.
  In our TracePro simulation, the dependency of the conversion efficiency with temperature is not considered because of the following. Phosphor conversion efficiency is dependent on two factors which are quantum efficiency and stoke shift efficiency. The Stokes-shift efficiency is only weakly dependent on temperature. The quantum efficiency decreases 0.117% per 1 °C increases in temperature^41^. In our experiments, the maximum temperature change observed is approximately 15 °C, and hence the change in the quantum efficiency is only 1.75%. To avoid complexity in the simulation, the change in phosphor conversion efficiency with temperature is not considered in view of the small change as mentioned above.
- As more heat is generated with the increase in the phosphor particles, the temperature of the phosphor layer increases with the amount of phosphor particles. Figure 15 shows our finite element analysis using ANSYS with the simulation parameters as in the work of Singh and Tan^5^. This is indeed consistent with the experimental observation in Fig. 14. The detail of the thermal analysis in our work will be described later.

When the amount of phosphor particle reaches a certain value, which is around 64 phosphor particles in this case for the given thickness, the overall light output decreases. This decrease can be attributed to the following:

- Increased scattering of the blue and yellow light by the presence of the densely distributed phosphor particles, and some of the light is scattered backward and cannot emit from the layer. Figure 12 shows our TracePro simulation of the distribution of the blue and yellow light vs. amount of phosphor particles. It is observed that the blue light decreases whereas yellow light increases as the number of phosphor particles increases, due to conversion and reflection. The % of the blue light rays reflected backward is increasing with the amount of phosphor particles. On the other hand, the yellow light rays increases due to conversion as the amount of particles increase, but the rate of its increase is decreasing due to the increasing amount of blue rays reflected backward instead of participating in the conversion process. When the number of particles go beyond 128, backward reflection of the yellow light rays also occurs. Hence, based on the TracePro simulation, we should see a shift of the white light toward warm light as the number of particles increases, and the rate of the increase in the light output is decreasing. Further increase in the amount of particles will results in a decrease in the total amount of light output. All the above mentioned is consistent with the experimental results shown in Figs 4 and 13.

**Figure 14:**
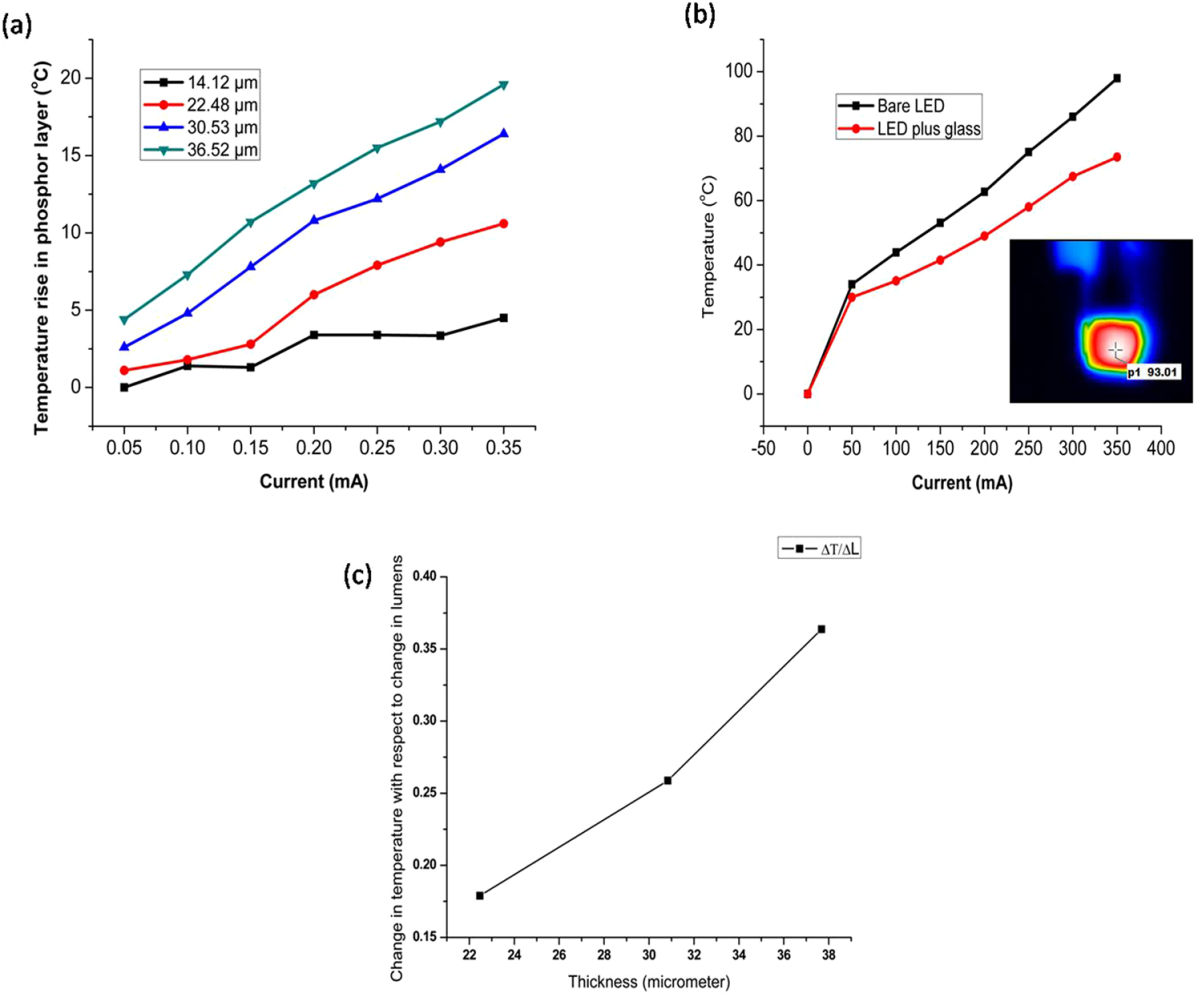
(**a**) Temperature rises in the phosphor layers of different thicknesses (the temperature change in the bare glass substrate is subtracted from the temperature of the samples with varying phosphor thickness). (**b**) Temperature rise in the bare LED and LED plus glass (no phosphor structure) vs current measured. Inset shows the thermal image taken for one of the samples. (**c**) Change in temperature per unit change in lumen for phosphor layers with different thickness at 250 mA driving current.

On the other hand, when the thickness of the phosphor layer increases for a given amount of phosphor particles, the lumen output increases as seen in Fig. 14(b). This is because the scattering become less as the particles are more dispersed.

In our experiments, the concentration of the phosphor content is kept constant. Hence, when our phosphor layer increases, the amount of phosphor particles increases, and its thickness also increases. The on-set of the decrease in the lumen output will therefore arrive when the effect of thickness increase becomes less than the effect of the increase in the amount of phosphor particles, and this is observed beyond 30 μm in our experimental samples. For different concentrations, the on-set phosphor layer thickness where lumen output begins to decrease should therefore be different, and one would expect there is a concentration where such decrease in lumen output will not occur. On the other hand, there will also be some range of concentration where such decrease in lumen output will occur even earlier. The dependency of this on-set thickness and phosphor concentration is an interesting topic but it is beyond the scope of this work.

Based on the above-mentioned discussion, the mechanism of the lumen output decrease with the phosphor layer thickness increase is most likely to be due to the light scattering and back reflection of light rays due to denser phosphor particles.

Besides the photometric and chromatic changes, our thermal analysis results show that the temperature rises in the phosphor layer due to the blue to yellow light conversion increases with an increase in phosphor layer thickness as shown in Fig. 14(a) for a given current as explained earlier. The temperature is measured using a high-Performance Thermo Image Camera with model no. P384A-20 at 14 um spectral range and sensitivity of less than 0.04 °C.

The data presented in Fig. 14(a) for temperature in samples with varying phosphor layer thickness is after subtracting the temperature of the blue LED combined with glass slide in order to show the actual average temperature rise in the phosphor layer. The bare LED and LED combined with glass temperature rise with respect to current are measured separately and they are shown in Fig. 14(b). It clearly suggests that the glass slide facilities heat dissipation to the ambient and this is the rationale for using the LED combined with glass slide in our Fig. 14(a) data as a base line reference.

The increase in the temperature of the phosphor layer is also higher for higher drive current as can be seen in Fig. 14(a), and this is expected since more conversion occur at higher drive current. As high temperature will lead to shorter LED lifetime, high lumen flux through thicker phosphor layer with more phosphor particles must be limited. In fact, when the thickness is beyond 30.53 μm, there is no gain in the lumen flux as can be seen in Fig. 2(a), but the temperature of the phosphor layer continues to increase, in addition to the shift of the light toward yellow as indicated in Fig. 13. Thus, we should limit the phosphor layer thickness to around 30 μm.

The increase in temperature for a unit increase in the light output intensity for different phosphor thickness is shown in Fig. 14(c). We can see that the rate of increase in temperature with the phosphor thickness is higher when the thickness is above 30 μm, and this is consistent with the conversion efficiency shown in Fig. 14(b).

When the phosphor layer thickness is 14.12 µm, the heat generated in the conversion process at the phosphor sites can be dissipated to the surrounding via glass slide as the layer does not have 100% coverage on the glass, and thus the temperature at phosphor sites is lower, and the rate of increase in temperature with the drive current is also smaller due to more effective heat dissipation in this case as compared to other higher phosphor layer thicknesses. Another reason is the lower amount of back scattering and absorption of light in the case of thinner phosphor layer (since the amount of phosphor particles are smaller).

When the phosphor layer becomes thicker, the temperature increase is higher due also to the less effective heat dissipation through the glass substrate and higher rate of back scattered and absorbed light. All the back scattered light will be absorbed either by the LED or the glass substrate. This absorbed light will be transformed into heat and thus increasing the overall temperature of the package. This is clearly observed in Fig. 14(a) which shows that the average rates of change of phosphor temperature with the drive current for phosphor layer thicknesses of 30.53 and 36.52 μm are the same, and that they are much higher than that for phosphor layer thicknesses of 14.12 μm. Knowing that the heat has to travel through the phosphor layer before it can be dissipated to the surrounding and glass substrate for thicker phosphor layer, the high rate of increase in temperature is expected.

The rate of temperature increase for the phosphor layer of 22.48 μm shows two different trends as seen in Fig. 14(a), indicating that when the heat generated is low at lower drive current, glass substrate and the surrounding are effective media to dissipate the heat, due probably to the conversion sites are near to the glass substrate at lower drive current. However, when the generated heat becomes higher and deeper into the phosphor layer at higher drive current, heat dissipation through the phosphor layer will have to come in, and the rate is a combination of the both the rates through glass substrate and the phosphor layer.

From the above discussion, one can see that while the lumen increases with phosphor layer thickness, its temperature also increases that shorten the lifetime of W-LEDs. Consequently, an optimal phosphor layer thickness should be determined such that one can have the maximum increase in the lumen with the minimum increase in the temperature.

**Figure 15:**
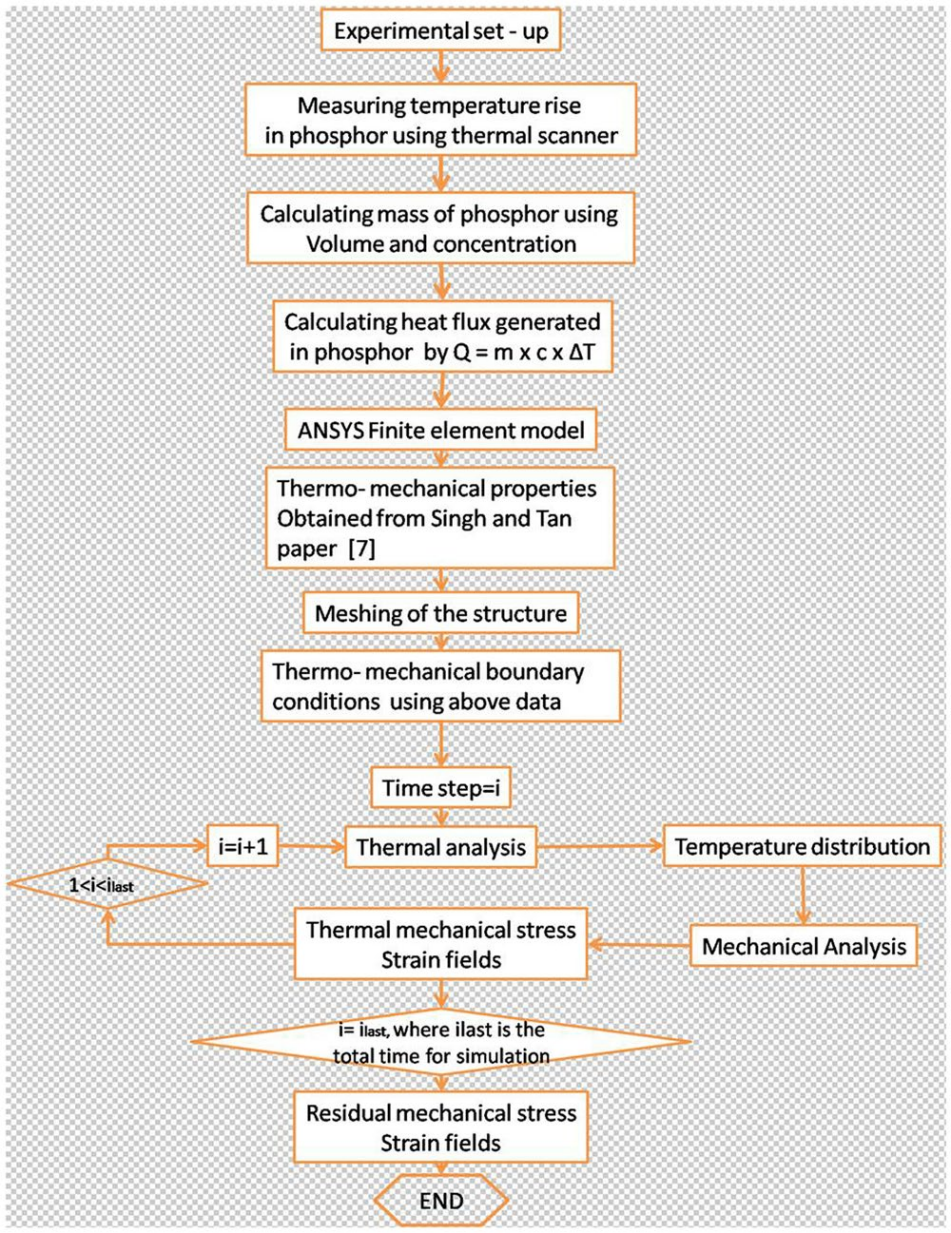
Flowchart for the ANSYS thermos-mechanical simulation to analyze the strain pattern and temperature flow in a packaged LED from our experimental results.

Although our work is very different from packaged LED, the results that obtained so far can be used for the design of encapsulated LED. To demonstrate an application of our work to the actual encapsulated LED package, we performed thermal analysis of a LED package and calculated the heat produced inside the LED package with different phosphor layer thickness. The phosphor is assumed to be placed on the LED chip and simulation is performed using ANSYS APDL as shown in the flow chart in Fig. 15.

**Figure 16:**
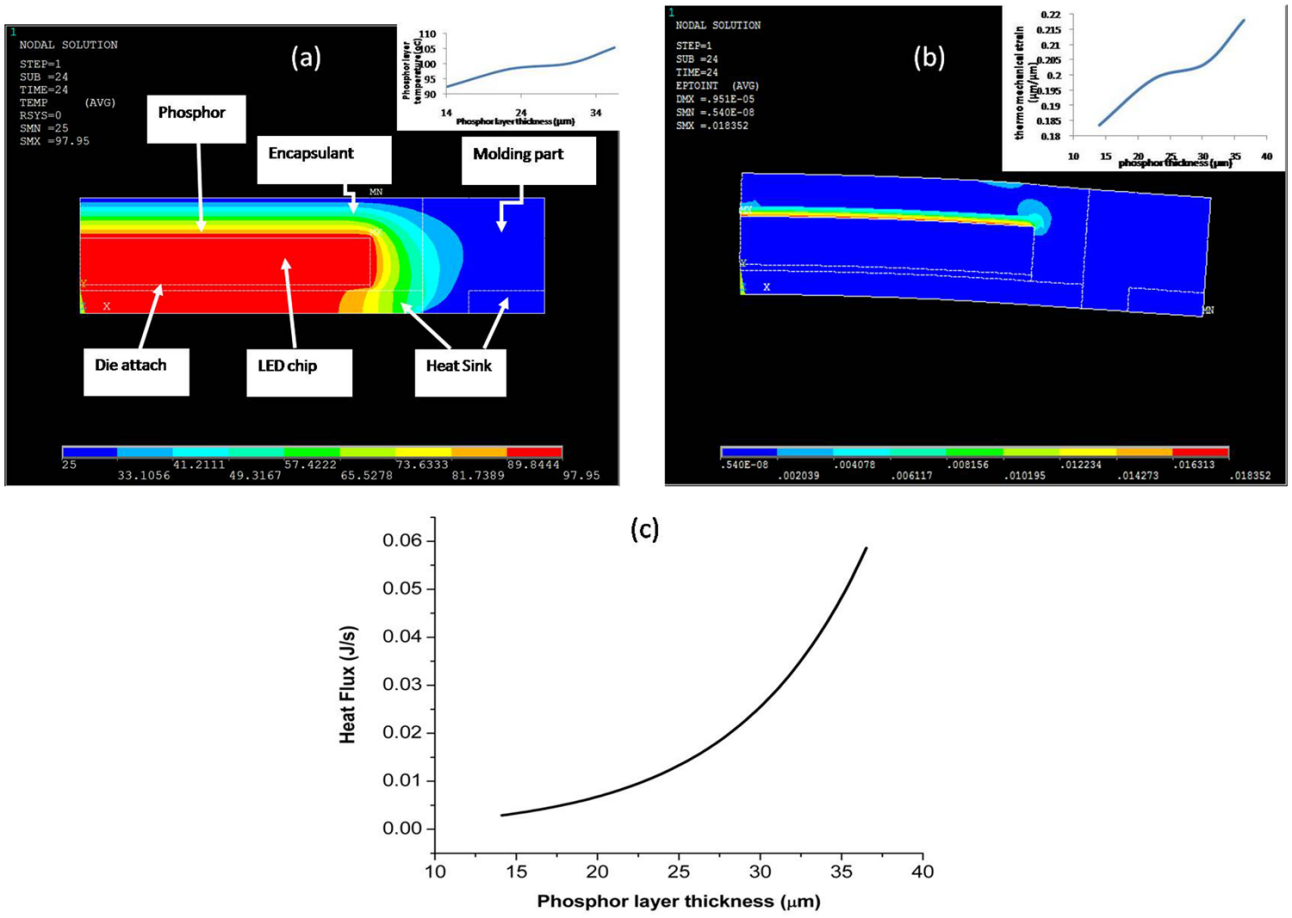
(**a**) ANSYS simulation of the temperature distribution of the encapsulated LED structure where phosphor is coated onto the blue LED dice which is powered at 0.35 A. The insert shows the temperature of phosphor increases with its thickness; (**b**) Thermo-mechanical strain distribution and the inset shows the increase in strain with varying phosphor thickness; (**c**) total heat flux accumulation in the LED package.

The ANSYS simulation structure is shown in Fig. 16(a) and properties are chosen from the work of Singh and Tan^5^. The LED temperature is given as 97.95 °C as measured during our experiments and the heat generated in the phosphor layer is calculated for the phosphor thickness by the well-known formulae Q = m × c × ΔT. Here Q, m, c and T represents heat flux, mass, specific heat and temperature generated in the phosphor layer.

Figure 16(a) shows the temperature distribution of the encapsulated LED and Fig. 16(b) shows the distribution of the corresponding thermo-mechanical strain in the package where a clear deformation is observed in the sample. The thermo-mechanical strain increases with the increase in the phosphor layer thickness as shown in Fig. 16(b).

From our ANSYS simulation, it is clearly observed that the total heat flux is increasing inside the package as the phosphor layer thickness is increased. Another important point which can be observed from Fig. 16(a) is that the rate of temperature increase is higher for higher thickness as compared to the thinner phosphor layer as the rate of increase of heat flux with thickness becomes higher at larger phosphor layer thickness. Beyond 30 $\mu m$, thermo-mechanical stress also increase sharply.

## Conclusion

Enhancement of lumen flux for white LEDs is always desired, and this can be achieved through increasing the drive current or the phosphor layer thickness. While these methods do increase the lumen flux, they also present some reliability impact to the W-LEDs. In this work, we examine the impact of phosphor thickness and phosphor concentration, and we found that there is a maximum phosphor layer thickness (for a given phosphor concentration) beyond which the lumen will not increase but the temperature will increase, render its effectiveness. All the possible causes for such reduction in light output are investigated in detail, and we found that it is the backscattering of light by the phosphor particles that results in the overall light output decrease. TracePro is employed to simulate the scattering of light and back reflection phenomenon to confirm the mechanism.

Our experimental results can also provide information for the temperature and thermo-mechanical stress in a packaged LEDs which is demonstrated in this work.
